# Preliminary report of an outbreak of SARS-CoV-2 in mink and mink farmers associated with community spread, Denmark, June to November 2020

**DOI:** 10.2807/1560-7917.ES.2021.26.5.210009

**Published:** 2021-02-04

**Authors:** Helle Daugaard Larsen, Jannik Fonager, Frederikke Kristensen Lomholt, Tine Dalby, Guido Benedetti, Brian Kristensen, Tinna Ravnholt Urth, Morten Rasmussen, Ria Lassaunière, Thomas Bruun Rasmussen, Bertel Strandbygaard, Louise Lohse, Manon Chaine, Karina Lauenborg Møller, Ann-Sofie Nicole Berthelsen, Sarah Kristine Nørgaard, Ute Wolff Sönksen, Anette Ella Boklund, Anne Sofie Hammer, Graham J. Belsham, Tyra Grove Krause, Sten Mortensen, Anette Bøtner, Anders Fomsgaard, Kåre Mølbak

**Affiliations:** 1 Statens Serum Institut, Copenhagen, Denmark; 2 Department of Veterinary and Animal Sciences, Faculty of Health and Medical Sciences, University of Copenhagen, Copenhagen, Denmark; 3 Department of Animal Health, Danish Veterinary and Food administration, Copenhagen, Denmark

**Keywords:** SARS-CoV-2, COVID-19, coronavirus, mink, Neovison vison

## Abstract

In June–November 2020, SARS-CoV-2-infected mink were detected in 290 of 1,147 Danish mink farms. In North Denmark Region, 30% (324/1,092) of people found connected to mink farms tested SARS-CoV-2-PCR-positive and approximately 27% (95% confidence interval (CI): 25–30) of SARS-CoV-2-strains from humans in the community were mink-associated. Measures proved insufficient to mitigate spread. On 4 November, the government ordered culling of all Danish mink. Farmed mink constitute a potential virus reservoir challenging pandemic control.

Until recently, Denmark was a leading producer of mink pelts. In June 2020, severe acute respiratory coronavirus 2 (SARS-CoV-2) began to spread among mink farms [1] and, along with infections in mink, infections in people connected to mink farms were detected. Whole genome sequencing (WGS) confirmed community spread of mink-associated SARS-CoV-2 strains (mink variant). We briefly describe the human outbreaks related to mink and the public health response.

## Epidemiological and laboratory investigations

Mink farms with SARS-CoV-2 circulating in mink, as well as people connected to mink farms (all residents on a mink farm production site or residing at the same address than a mink-farm owner; employees living outside mink farms could not be identified), were identified by four different approaches. (i) In Denmark, human SARS-CoV-2 infections (confirmed by PCR) are reportable by laboratories, and laboratory data are electronically submitted to Statens Serum Institut (SSI). Case reports were linked to an address register and a database of mink farm owners, provided by the Danish Veterinary and Food Administration (DVFA), thereby identifying human cases residing on mink farms. This group was encouraged to take a weekly PCR-test, in order to prevent spread of infection to the mink. (ii) Contact tracing of human cases, carried out by the Danish Patient Safety Authority, enabled identification of case patients with any connection to mink production. (iii) A surveillance programme established by DVFA, based on submission of samples from dead mink from all mink farms to SSI. (iv) Reporting of clinical signs in mink by veterinarians. Infection in mink on farms was confirmed after sampling by DVFA and submission of samples to SSI for SARS-CoV-2-testing by PCR and antibody test.

Estimates of the cumulative regional incidence rates per 100,000 population of human mink variant strain infections in the community were calculated. This was done by adding the weekly estimates (the number of all SARS-CoV-2-positive samples multiplied by the frequency of the mink variant strain among sequenced samples) and their variances, to take into account the variability of sample size and prevalence of mink variant strain over time.

Maps were created with R version 4.0.2 [2].

WGS was undertaken on virus samples from mink and human cases [1]. The Danish sequencing programme was established early during the coronavirus disease (COVID-19) pandemic with collaborators at SSI and at Aalborg University. The consortium is described at https://www.covid19genomics.dk. The sequencing technology was Oxford nanopore. The sequencing consortium receives samples from all laboratories in Denmark, and thereby characterises an unbiased sample of strains. As the typing efforts were intensified as a response to the outbreak, we stratified for week and region in the statistical analysis in order to obtain valid estimates at the population level. A total of 13,355 samples (20% of all 65,872 PCR-positive samples) were sequenced during the period between 10 August and 29 November 2020.

## Ethical statement

No ethical approval was required for this register-based study.

## Outbreak description

**Figure 1:**
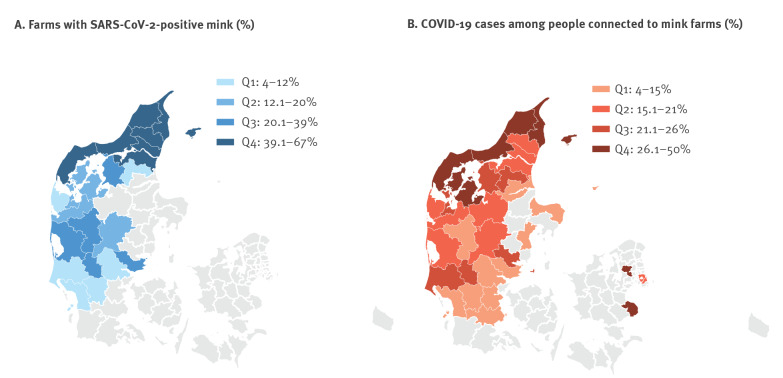
Proportion by municipality of (A) farms with SARS-CoV-2-positive mink among mink farms and (B) COVID-19 cases among people identified as connected to mink farms^a^, Denmark, 10 August–29 November 2020^b^

Source: The original shapefile of Danish municipalities is from the Database of Global Administrative Areas, GADM (www.gadm.org), version 2.5, July 2015.

Q1-4: quartile percent range; SARS-CoV-2: severe acute respiratory syndrome coronavirus 2.

^a^ As representatives for the occupational risk.

^b^ The three farms with SARS-CoV-2-infected mink in June were incidentally detected. During July the ad-hoc surveillance became operational. Only data from 10 August onwards, which were collected once the surveillance was in place are included in the Figure.

**Figure 2:**
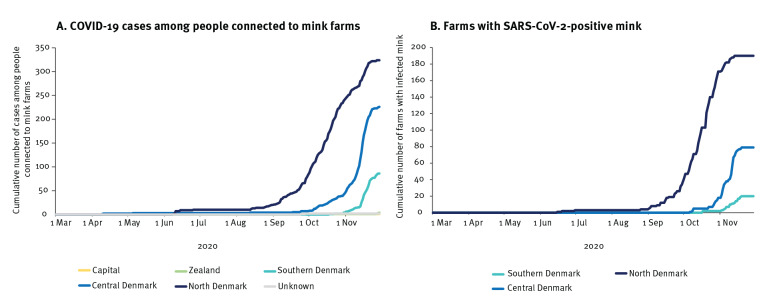
Cumulative numbers of (A) cases of COVID-19 among people identified as connected to mink farms^a^ and (B) farms with SARS-CoV-2-positive mink, by geographical region, Denmark, June–November 2020

COVID-19: coronavirus disease; SARS-CoV-2: severe acute respiratory syndrome coronavirus 2.

^a^ As representatives for the occupational risk.

From 8 June to the end of November, SARS-CoV-2 was detected in mink from 290 (25%) of 1,147 Danish mink farms, with the highest proportion of affected farms in North Denmark Region (48%) (190/394) [3]. The epidemic started in Northern Jutland (North Denmark Region), and spread to other areas of Jutland during October. Overall, 643 of 3,319 (19%) people identified as being connected to mink farms became infected. The attack rate was highest in North Denmark where 30% (324/1,092) became infected. Figure 1 shows the situation in the country with, by municipality, the proportion of farms with SARS-CoV-2-positive mink, as well as the proportion of COVID-19 cases among people found connected to mink farms. Figure 2 shows development over time for farms with SARS-CoV-2-positive mink and connected human cases, by region.

To maintain patient confidentiality, the municipalities of Læsø and Frederikshavn, and the municipalities of Silkeborg and Ikast –Brande, were merged. In the municipalities of Egedal and Stevns (within Zealand), only a few people were connected to mink production, and therefore the one and four positive samples, respectively, constituted large proportions of this group of people.

A mink variant SARS-CoV-2 strain, which had the spike protein change Y453F, was initially observed in an outbreak in mink and humans in June 2020 [1]. The proportion of mink variant strains [4] among all sequenced samples was estimated separately by week and region, in order to adjust for variations before calculating the cumulative incidence (Table 1). Approximately 4,000 human cases were estimated to be infected with a mink variant. The proportion of mink variant strains varied between regions, with the highest average proportion during the period of August to November (27%) found in North Denmark Region (Table 1). In this region, human COVID-19-cases identified caused by a mink variant constituted 53% (101 of 190 sequenced samples; 95% confidence interval (CI): 46–60) in weeks 41–42 but decreased to 26% (206 of 780 sequenced samples; 95% CI: 23–30) in weeks 46–48. Mink variant strains also became common in the regions of Central Denmark and Southern Denmark during weeks 46–48, as the number of farms with infected mink increased in these areas. In the Capital Region, 45 human cases infected with the mink variant, within 42 different households were identified and, in the Zealand Region, 12 human cases of mink variant infections, within 10 different households were identified. Estimates of the cumulative incidence can be seen from Table 1.

**Table 1:** Regional overview of human COVID-19 cases and cases caused by mink variant strains, Denmark, 10 August–29 November 2020

Region	Total number of confirmed human COVID-19 cases (PCR)	Total number of sequenced samples (WGS)^a^	Number of confirmed human cases caused by mink variant strains	Adjusted proportion (%) of cases caused by mink variant strains^b^		Cumulative incidence of human COVID-19 cases caused by mink variant strains per 100,000 population^c^	
				%	95% CI	Cases	95% CI
North Denmark	5,159	1,942	498	27	25–30	239	216–262
Central Denmark	12,953	2,426	259	12	11–13	117	104–130
Southern Denmark	9,613	2,090	81	5	4–6	40	31–48
Zealand	7,337	1,425	12	1	0–2	10	4–16
Capital	30,810	5,472	45	1	1–1	15	11–19

CI: confidence interval; COVID-19: coronavirus disease; WGS: whole genome sequencing.

^a^ Samples for WGS were selected by convenience sampling among the total number of confirmed human COVID-19 cases. An analysis of representativeness showed that the proportion of samples from human cases connected to mink production were similar to the population in general at the regional level (chi-squared p > 0.66 for North, Central, Southern and Capital regions, and p = 0,176 for Zealand Region).

^b^ Estimates of the proportions of COVID-19 cases with mink variant strains in the community were derived by sequencing SARS-CoV-2-positive human samples to detect those with the mink variant strain. Proportions of cases with mink variants were calculated by week, and adjusted for weekly variations, in the proportion of sequenced samples. WGS results were delivered on a weekly basis.

^c^ Regional population size of the last quarter of 2020, Statistics Denmark.

## Cluster 5 variant

We identified 35 substitutions (excluding D614G change) and four deletions in the spike protein of SARS-CoV-2 among variants co-circulating in mink and humans in Northern Jutland between June and November 2020. A mink-associated SARS-CoV-2 variant with a combination of changes in the spike protein (H69 and V70 deletion, Y453F, D614G, I692V, M1229I), not previously observed, was named ‘cluster 5’ and was found in 12 human cases and in mink on five mink farms in Northern Jutland, from August to September 2020. Preliminary findings suggested that there might be a lower capability of antibodies from convalescent patients to neutralise this variant [5]. As at 1 February 2021, we assess that the cluster 5 variant is no longer circulating among humans in Denmark.

## Public health response

Denmark has followed a number of different strategies in order to mitigate the spread of SARS-CoV-2 in mink farms and to prevent spillover to the human population and spillback from humans to mink, Table 2. After the first three farms with SARS-CoV-2-positive mink were identified and animals in these farms were culled in June, a policy, based on surveillance in the human–animal interface, enhanced biosecurity, and use of personal protective equipment, was adopted. This policy proved insufficient to stop the extensive spread of infections among mink farms, and the subsequent spread of mink-associated strains to the communities. The main reason for this seems to be late detection of farms with SARS-CoV-2-positive mink, i.e. at the time of detection, the infection was widespread within the farm, the mink tested antibody positive and persons related to the farms were already infected [1,6].

**Table 2:** Summary of control measures to mitigate the spread of SARS-CoV-2 at the human–mink interface in Danish mink farms, Denmark, June–November 2020

Period (2020)	Surveillance and control measures in mink farms	Comments
June	Culling of mink in the first three farms with SARS-CoV-2-positive mink, establishment of a surveillance programme with sampling from dead mink on all farms every 3 weeks, surveillance of PCR test results from people connected to mink farms, and sampling from mink in 125 randomly selected farms for PCR testing.	On a precautionary principle, and based on experiences from the Netherlands, animals in the first three farms were culled.
July–September	Containment strategy without culling, based on enhanced biosecurity, use of personal protective equipment in mink farms and enhanced surveillance of dead mink.	From 4 July to 11 August, no additional farms with SARS-CoV-2-positive mink were detected by surveillance. The screening of 125 randomly sampled farms did not reveal farms with SARS-CoV-2-positive mink, and no SARS-CoV-2-positive mink farm residents were observed. From mid-August SARS-CoV-2 re-emerged in mink farms.
October	Decision to cull mink in farms with infected mink as well as mink in neighbouring (i.e. within a distance of 7.8 km) mink farms. Surveillance programme intensified to comprise sampling of dead mink from herds within the 7.8 km zones twice per week until culling.	In September, the number of farms with SARS-CoV-2-positive mink showed a marked increase, and the incidence of human COVID-19 cases increased in parallel. The zone of 7.8 km was established based on an analysis of minimum distance to the nearest farm with SARS-CoV-2-positive mink [14]. These measures did not prevent further spread to other mink farms and humans.
November	Decision to cull all mink in Denmark and temporarily halt mink production.	A risk assessment was issued by Statens Serum Institut on 3 November 2020. This assessment stated that during the COVID-19 pandemic, an ongoing production of mink represents a hazard to human public health [7].

COVID-19: coronavirus disease; SARS-CoV-2: severe acute respiratory syndrome coronavirus 2.

On 1 October, when it was decided to cull mink on farms with SARS-CoV-2-positive mink and mink in neighbouring farms situated within a distance of 7.8 km, there were 41 farms with SARS-CoV-2-positive mink, primarily in the North Denmark Region (Figure 2,panel B). A month later, there were 207. Overall, by the end of October, there was a continued geographical spread and an increasing number of farms with SARS-CoV-2-positive mink in several municipalities over most of the peninsula of Jutland (Figure 1). On 4 November 2020, the government decided to cull all farmed mink in Denmark. Despite the intensive culling activity for either destruction or pelting, the number of farms with SARS-CoV-2-positive mink reached 290 before almost all mink were culled by 27 November. This decision was made following lessons learned, i.e. it had not been possible to prevent the spread of infection from humans to animals, from farm to farm, or from animals to humans, and thereby mink contributed substantially to the ongoing transmission of SARS-CoV-2 in the western part of Denmark [7].

On 5 November 2020, a partial lockdown was introduced in the seven municipalities with the highest number of farms with SARS-CoV-2-positive mink in Northern Jutland, and a mass-testing of the human population was initiated. On 19 November 2020, restrictions were lifted due to decreased incidence and the absence of new cases of the cluster 5 variant.

By 25 November 2020, mink on all 290 farms with SARS-CoV-2-positive mink, and mink on farms within an assigned zone of 7.8 km from the nearest infected farm, had been culled. The few remaining animals were culled during December and January when legislation was passed and enforced to ban mink farming in Denmark until 31 December 2021, including import and export of live mink.

## Discussion

Spread of SARS-CoV-2 in mink farms and at the human–animal and/or animal–human interface has been reported from several countries, including Canada, France, Greece, Italy, Lithuania, the Netherlands, Spain, Sweden and the United States [8-10].

In the current study, people in households located on Danish mink farms have been identified as having an occupational risk for SARS-CoV-2 due to direct or indirect exposure in the farm environment. It can be assumed that at least one member of a mink farm household had regular access to the animals and farm area. Moreover, from our surveillance data, the occurrence of SARS-CoV-2 in people connected to mink farms seemed to be closely related to the occurrence of the virus in mink. It should be noted, however, that it was not possible to identify employees living outside a mink farm. These factors may have led to an underestimation of the occupational risk of mink farming in areas with SARS-CoV-2-positive mink.

In addition, we observed from June 2020 the emergence of the mink variant of SARS-CoV-2, with transmission of this both in mink and people connected to mink farms. These strains also spread further in the community [1]. At least 90 human cases were linked to the June outbreak caused by the mink variant, including residents and staff at a nursing home and participants in a bus trip to Bornholm [11,12]. In the second wave, starting in August, community spread of the mink variant was seen in the municipalities with highest numbers of farms with SARS-CoV-2-positive mink, and at the peak of the epidemic more than half of the strains sequenced from human samples in North Denmark Region were mink-associated. This spread was of substantial public health concern, since it contributed to the emergence of SARS-CoV-2 in an area of Denmark that hitherto had very few COVID-19-cases, relative to urban areas of Denmark.

Until June 2020, Denmark accounted for ca 40% of the world production of mink furs and had a mink population of some 17 million animals. Approximately 25% (290/1,147) of the Danish farms had infected mink before the culling ended, corresponding to ca 3–4 million infected animals compared with less than 300,000 humans being infected, based on serological studies (3.1% positive blood donors as of first week of December 2020 [13]). A massive and susceptible animal population serves as a threat for infection and transmission, and for viral adaptation and potential immune escape that may spill back into the human population. The Danish experiences are unique because of the magnitude of the Danish mink production. However, other countries with farmed mink may well experience similar risks.

The Danish experience calls for a global and coordinated One-Health approach to understand and mitigate the risk that farmed mink may pose for the control of the pandemic and to act accordingly.
